# Nucleus accumbens D1-receptors regulate and focus transitions to reward-seeking action

**DOI:** 10.1038/s41386-022-01312-6

**Published:** 2022-04-27

**Authors:** Laura L. Grima, Marios C. Panayi, Oliver Härmson, Emilie C. J. Syed, Sanjay G. Manohar, Masud Husain, Mark E. Walton

**Affiliations:** 1 Department of Experimental Psychology, University of Oxford, Oxford, UK; 2 Medical Research Council Brain Network Dynamics Unit, University of Oxford, Oxford, UK; 3 Nuffield Department of Clinical Neurosciences, University of Oxford, Oxford, UK; 4 Wellcome Centre for Integrative Neuroimaging, University of Oxford, Oxford, UK; 5 Present Address: Janelia Research Campus, Howard Hughes Medical Institute, Ashburn, VA USA; 6 Present Address: National Institute on Drug Abuse, Biomedical Research Center, 251 Bayview Boulevard, Suite 200, Baltimore, MD 21224 USA

**Keywords:** Reward, Motivation

## Abstract

It is well established that dopamine transmission is integral in mediating the influence of reward expectations on reward-seeking actions. However, the precise causal role of dopamine transmission in moment-to-moment reward-motivated behavioral control remains contentious, particularly in contexts where it is necessary to refrain from responding to achieve a beneficial outcome. To examine this, we manipulated dopamine transmission pharmacologically as rats performed a Go/No-Go task that required them to either make or withhold action to gain either a small or large reward. D1R Stimulation potentiated cue-driven action initiation, including fast impulsive actions on No-Go trials. By contrast, D1R blockade primarily disrupted the successful completion of Go trial sequences. Surprisingly, while after global D1R blockade this was characterized by a general retardation of reward-seeking actions, nucleus accumbens core (NAcC) D1R blockade had no effect on the speed of action initiation or impulsive actions. Instead, fine-grained analyses showed that this manipulation decreased the precision of animals’ goal-directed actions, even though they usually still followed the appropriate response sequence. Strikingly, such “unfocused” responding could also be observed off-drug, particularly when only a small reward was on offer. These findings suggest that the balance of activity at NAcC D1Rs plays a key role in enabling the rapid activation of a focused, reward-seeking state to enable animals to efficiently and accurately achieve their goal.

## Introduction

The balance of dopamine transmission plays a key role in mediating the efficacy of reward-guided behavior [1–4]. Disrupted dopamine transmission, particularly in the nucleus accumbens core (NAcC), reduces the likelihood of responding to reward-associated cues and disrupts the willingness to persist with instrumental responses [5–9]. Conversely, hyperdopaminergic states can result in aberrant and impulsive reward pursuit [10–13]. Nonetheless, the precise relationship between reward expectation, dopamine transmission, and behavioral control remains unclear.

It is well established that reward-associated cues drive changes in dopamine activity proportional to the anticipated future benefit [14–17]. One prominent idea is that this information provides a signal that can be used to update value estimates and thus influence the speed and accuracy of decisions in that state [18]. However, there is accumulating evidence that dopamine activity is itself shaped by action demands [19–23]. Therefore, an alternative is that dopamine provides a Pavlovian signal to elevate responding based on reward expectations [4, 24–29]. Accordingly, changes in dopamine would primarily affect the likelihood and/or vigor of reward-seeking actions. A third possibility is that dopamine might not only regulate action likelihood but also the precision of reward-seeking actions based on the potential benefit that could be accrued. Reduced reward sensitivity in Parkinson’s Disease patients has been attributed partly to an increase in the cost of ensuring actions are precisely executed [30]. We here collectively term the facility to ensure goal-directed sequences are performed rapidly, repeatedly, and successfully as behavioral “focus”. Behavioral focus in the form of cognitive control may also be governed by dopamine [31, 32].

One method to adjudicate between these accounts is to compare how manipulating dopamine transmission affects response efficacy and vigor in situations when animals need either to make or withhold a response to gain different amounts of reward. To do this, we trained rats on a symmetrically rewarded Go/No-Go task [22, 33] and investigated the effects of pharmacological stimulation and blockade of dopamine receptors, first systemically and then locally in the NAcC. We focused on the role of D1-like receptors (D1Rs) as these are believed to play an important role in mediating how phasic changes in dopamine influence the downstream activity of striatal medium spiny neurons (MSNs) [34–39].

## Materials and methods

**Fig. 1:**
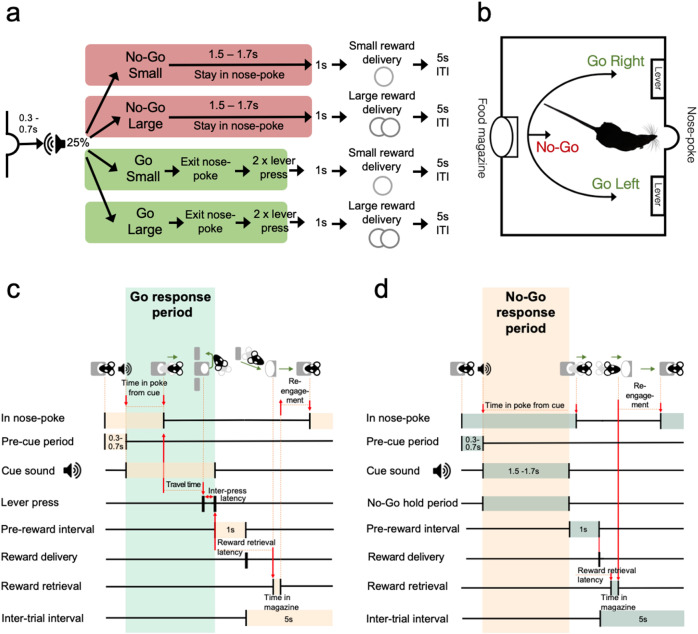
Schematics illustrating the sequence of events and associated metrics in correctly executed Go and No-Go trials. **a** Schematic of task trial types. All trials were initiated by the animal entering the nosepoke, which after a short delay resulted in the presentation of one of four auditory cues. Here, colored shading indicates when auditory cues remained on. The identity of the cue instructed rats to either leave the nosepoke and respond on the left or right lever, each of which was associated with either a small or large reward (side fixed for each animal, counterbalanced across animals) (Go Small or Go Large) or to remain in the nosepoke for the holding period to gain either small or large reward (No-Go Small or No-Go Large). Correct trials were followed by a 5-s inter-trial interval (ITI). **b** Schematic of the operant chamber layout. **c** Measured latencies in Go trials. Orange shaded areas between task events indicate the time between events. Arrows indicate the start and end of stated behavioral latencies. Green shading indicates Go trial response period, from leaving the nosepoke to completing two lever presses successfully. **d** Same as in (**c**) but for No-Go trials. Here, green shading indicates task events and orange shading indicates the response period, in which mice were required to stay in the nosepoke. Arrows again indicate behavioral latencies.

All procedures were carried out in accordance with the UK Animals (Scientific Procedures) Act (1986). A total of 25 adult male Sprague Dawley rats (Harlan, UK), split into two cohorts (cohort 1 = 11 rats, cohort 2 = 14 rats) were used in the reported studies (Supplementary Table S1). Rats were trained on an operant Go/No-Go task that required them either to make (Go) or withhold (No-Go) action in order to gain either a small or large reward [22, 33] (Fig.1; see Supplementary Info).

### Performance measures

Response accuracy was a primary behavioral metric. On Go trials, errors were divided into selection of the incorrect lever (“WRONG LEVER”) or failing to make a response in the 5 s during a cue (“RESPONSE OMISSION”). No-Go errors resulted from exiting the nosepoke before the end of the cued holding period (“PREMATURE EXIT”), and were divided into those occurring in the first (“EARLY”, <800 ms) or second (“LATE”, >800 ms) half of the No-Go holding period [33]. Head exits made during the pre-cue period were also recorded (“ABORTED” trials).

Key task latencies in all successful trials (Fig. 1c, d) included: (a) TIME IN POKE FROM CUE: cue onset to nosepoke exit, (b) TRAVEL TIME (Go trials only): time from nosepoke exit to first lever press; and (c) REWARD RETRIEVAL: reward delivery to magazine entry. In addition, we calculated (d) RE-ENGAGEMENT: latency from magazine entry on a successful trial to re-entering the nosepoke. Analysis of animals’ trajectories was performed using the DeepLabCut toolbox [40] (see Supplementary Info). A summary of these performance and latency measures can be found in the Supplementary Methods (Supplementary Table S2).

### Pharmacological challenges

A full description of pharmacological compounds and doses used can be found in Supplementary Info (Supplementary Table S3). Drugs included a D1R agonist, D1R antagonist, D2R agonist, and D2R antagonist. The D1R drugs were given both systemically and locally (full details can be found in Supplemental Methods; histology for cannulae placements in Supplementary Fig. S6), whilst D2R drugs were given systemically. Drug administration sessions were separated by at least one treatment-free training day to ensure a return to baseline performance and complete washout of the drug.

## Results

### Reward size and action requirements shape baseline performance on the task

**Fig. 2:**
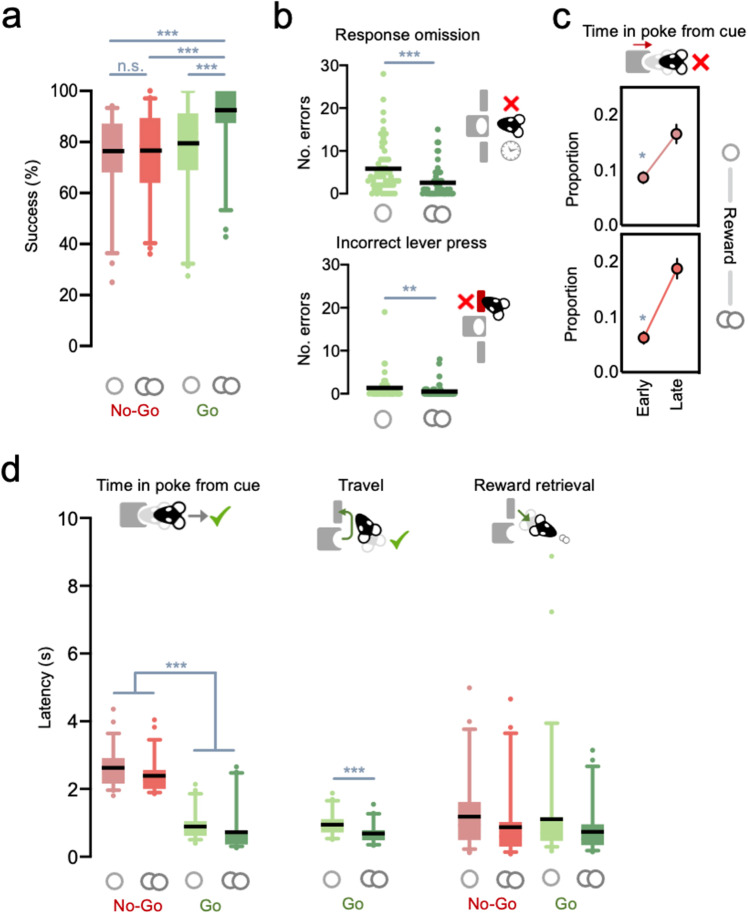
Go/No-Go task baseline performance. **a** Animals’ performance in vehicle sessions by session split by trial type (red: No-Go; green: Go; lighter shades denote small reward trials and darker shades denote large reward trials). Solid lines indicate the mean, box extends from 25th to 75th percentiles, whiskers indicate 5th and 95th percentiles. Pairwise comparisons: Go Large vs. Go Small/No-Go Small/No-Go Large: all *p* < 0.001; all other comparisons n.s., *p* > 0.4. **b** Top: total response omission errors per session. Pairwise comparison: Go Small vs. Go Large: *p* < 0.001. Bottom: total incorrect lever press errors per session. Pairwise comparison: Go Small vs. Go Large: *p* = 0.006. Interaction: wrong lever small vs. wrong lever large: *p* < 0.001; Response omissions small vs. response omissions large: *p* = 0.010. **c** Mean proportion of times spent in the nosepoke across error No-Go trials in which animals exited early (<800 ms) or late (>800 ms) when a small (upper) or large (lower) reward was on offer. Pairwise comparisons: early small vs. early large: *p* = 0.008; late small vs. late large: n.s., *p* = 0.134. **d** Mean latencies to complete key task events in correct trials split by trial type. ***p < 0.001, **p < 0.01, *p < 0.05.

We first sought to characterize how reward and action demands shaped Go/No-Go performance. Animals on average achieved >75% success rate across trial types (Fig. 2a). However, reward size only influenced response accuracy on Go but not on No-Go trials (action × reward interaction: *F*_(1,56)_ = 19.455, *p* < 0.001).

On Go trials, response omissions were more frequent than wrong lever presses (Fig. 2b; main effect of error type: *F*_(1,56)_ = 35.183, *p* < 0.001), though the occurrence of both errors was decreased when a large reward was on offer (main effect of reward: *F*_(1,56)_ = 25.374, *p* < 0.001; error type × reward interaction: *F*_(1,56)_ = 7.834, *p* = 0.007). On No-Go trials, premature responses were overall most likely in the “late” period (Fig. 2c; main effect of No-Go period: *F*_(1,56)_ = 43.806, *p* < 0.001). Although reward size did not change the total number of No-Go errors, the prospect of a large reward significantly decreased inappropriate responses “early” but not “late” in the holding period (period × reward interaction: *F*_(1,56)_ = 6.040, *p* = 0.017). Behavior on Go and No-Go trials was also faster when a large reward was on offer, resulting in reduced time in the poke, travel time (on Go trials), and reward retrieval latencies (main effect of reward: all *F* > 21.17, *p* < 0.001) (Fig. 2d).

Importantly, although animals in the cannulated cohort had on average slightly lower success rates on all trial types (main effect of cohort: *F*_(1,56)_ = 6.102, *p* = 0.017), there was no difference across cohorts on almost all other measures (all main effects or interactions with cohort: *F* < 2.6, *p* > 0.1; except for cohort × reward for time in poke from cue (*F*_(1,56)_ = 4.699, *p* = 0.034), though even here post-hoc tests showed no difference between cohorts, both *p* > 0.2). Taken together, this demonstrates that baseline behavior is strongly and consistently regulated by action requirements and reward size.

### Global D1Rs regulate action initiation and the vigor of actions distal to reward

We next investigated what role global stimulation or blockade of D1Rs plays in regulating appropriate action restraint and action initiation for future reward.

#### No-Go trials

**Fig. 3:**
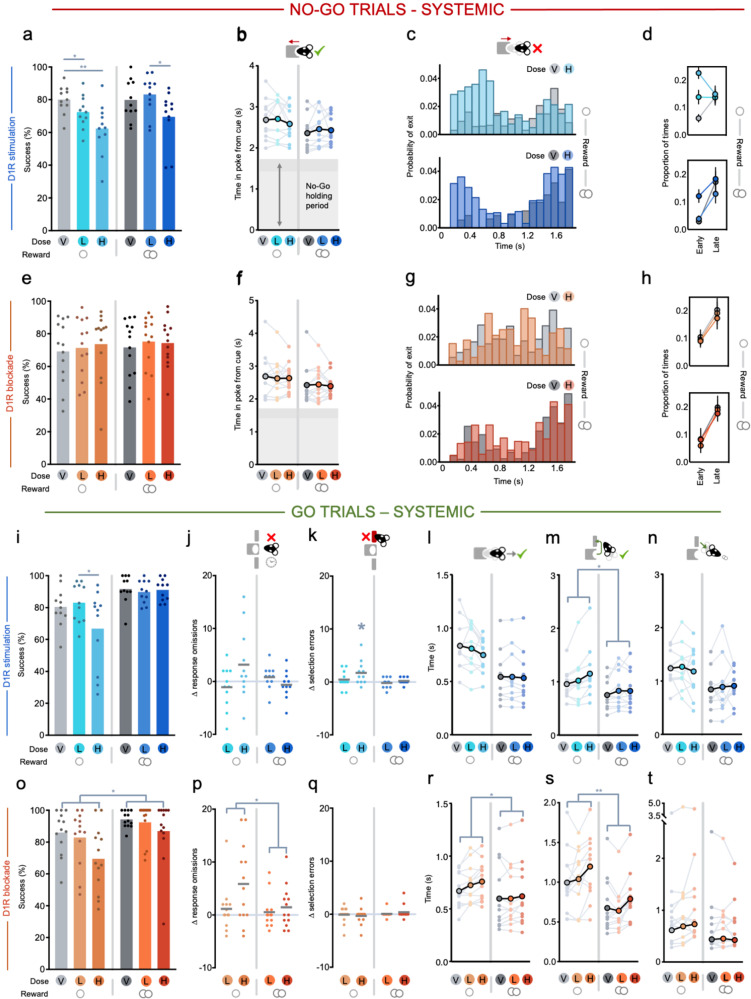
Effects of systemic D1R stimulation (SKF-81297) or blockade (SCH-23390) in No-Go and Go trials. V vehicle, L low dose, H high dose. Single circle indicates a small reward condition, double circle indicates a large reward condition. **a**, **b** Effects of D1R stimulation split by small (left) and large (right) reward No-Go trials on **a** success rate and **b** time in nosepoke from cue onset in successful trials. For **b**, analysis of pairwise comparisons due to significant drug × reward interaction: vehicle small reward vs. large reward: *p* = 0.005, low dose small reward vs. large reward: *p* = 0.012, high dose small reward vs. large reward: *p* = 0.071. Darker shading reflects the jittered No-Go holding period. **c** Mean probability histogram of time in nosepoke in failed small (upper) and large (lower) reward No-Go trials for vehicle (gray) or high dose (blue) manipulations, calculated as a probability over all head exit times. **d** Mean proportion of times spent in the nosepoke across trials in which animals exited early (<800 ms) or late (>800 ms) when a small (upper) or large (lower) reward was on offer. Pairwise comparisons: early period vehicle vs. low dose: *p* = 0.003, vehicle vs. high dose: *p* < 0.001; late period, all *p* > 0.5. **e**–**h** Same as in (**a**–**d**) but for systemic D1R blockade. **i**–**n** Effects of local D1R stimulation split by small (left) and large (right) reward Go trials on **i** success rate, **j** response omission errors (relative to vehicle session), **k** lever selection errors (relative to vehicle session), **l** latency to leave the nosepoke after Go cue onset, **m** latency from nosepoke exit to first lever press, **n** and latency from trial completion to entering the food magazine to retrieve the reward. **o**–**t** Same as in (**i**–**n**) but for systemic D1R blockade. **p < 0.01, *p < 0.05.

Systemic administration of a D1R agonist SKF-81297 had no influence on rates of aborted trials during the pre-cue period (main effect of drug: *F* < 0.5, *p* > 0.6, data not shown). However, it substantially impaired performance in No-Go trials (Fig. 3a; main effect of drug: *F*_(2,20)_ = 14.911, *p* < 0.001; drug × reward interaction: *F*_(2,20)_ = 3.467, *p* = 0.051). As can be observed in Fig. 3c, D1R stimulation selectively increased inappropriate action initiation only “early” in the No-Go hold period (Fig. 3d; drug × error period interaction: *F*_(2,20)_ = 7.780, *p* = 0.003; note, this is the opposite to the effect of reward size reported earlier in Baseline). However, on correctly performed No-Go trials, D1R stimulation did not change the overall speed of initiation or reward collection latency, although it did alter the difference between initiation latencies in small and large reward trials (Fig. 3b; drug × reward interaction: *F*_(2,20)_ = 7.264, *p* = 0.004; main effect of drug n.s., *F* = 0.473, *p* = 0.630).

By contrast, systemic administration of a D1R antagonist SCH-23390 had no significant effect on No-Go performance (Fig. 3e; no main effect of drug, reward, or interaction: all *F* < 0.6, *p* > 0.4) or response latencies (Fig. 3f–h; all *F* < 1.0, *p* > 0.4). These results from No-Go trials demonstrate that global stimulation, but not blockade, of D1Rs strongly regulates rapid inhibition of action.

#### Go trials

Unexpectedly, both global D1R stimulation and D1R blockade impaired performance on Go trials. Systemic administration of the D1R agonist reduced success rates selectively on Go Small trials at the highest dose (Fig. 3i; drug × reward: *F*_(2,20)_ = 4.135, *p* = 0.031). This was caused not only by a numeric increase in response omissions on Go Small trials (Fig. 3j; drug × reward: *F*_(2,20)_ = 3.346, *p* = 0.056), but also by a small but reliable increase in the number of wrong lever errors on Go Small trials (Fig. 3k; drug × reward: *F*_(2,20)_ = 4.515, *p* = 0.024). Although the D1R agonist numerically speeded animals’ latency to exit the start poke on small reward trials (Fig. 3l; *F*_(2,20)_ = 2.775, *p* = 0.086), it slowed travel times to the correct lever (Fig. 3m; main effect of drug: *F*_(2,20)_ = 6.331, *p* = 0.007) despite not having an effect on the subsequent latency to collect the reward (Fig. 3n; no main effect of drug or interaction: both *F* < 0.6, *p* > 0.5). Subsequent trial re-initiation latencies after success were also slower (main effect of drug: *F*_(2,20)_ = 11.954, *p* < 0.001).

The D1R antagonist also caused a dose-dependent reduction in Go trial success (Fig. 3o; main effect of drug: *F*_(2,24)_ = 7.015, *p* = 0.004; drug × reward interaction n.s, *F* < 1.1, *p* > 0.3). However, this was driven by increased response omissions (Fig. 3p; main effect of drug: *F*_(2,24)_ = 6.846, *p* = 0.004; drug × reward interaction, *F* < 2.9, *p* > 0.07) and there was no effect on the ability to select the correct lever (Fig. 3q; no main effect or interaction of drug: *F* < 0.9, *p* > 0.4). D1R blockade also slowed latencies, but this was evident for all Go trial actions aside from direct approach to the food magazine and was largely unaffected by reward size (Fig. 3r–t; time in poke, travel time, re-engagement latencies: main effect of drug: all *F* > 8.60, *p* < 0.003; drug × reward interaction: all *F* < 2.91, *p* > 0.07; reward retrieval: no main effect or interaction with drug: both *F* < 2.1, *p* > 0.15).

Therefore, both global stimulation and blockade of D1Rs impaired Go trial performance, but there was again an asymmetric effect of the two manipulations. D1R stimulation disrupted animals’ ability to efficiently select and execute the correct action. By contrast, D1R blockade markedly increased response omissions and slowed all actions other than reward retrieval. Moreover, this influence of D1Rs on time in poke from cue, restraint, and vigor appeared specific to this receptor, as systemic administration of a D2R agonist or antagonist caused distinct effects on performance (Supplementary Info, Supplementary Text 1 and Supplementary Fig. S1).

### D1Rs in NAcC selectively shape action likelihood and focus

The first experiments demonstrated a key selective role for D1Rs in the rapid modulation of action restraint and initiation. As our previous study had demonstrated a close relationship between fast, transient increases in dopamine levels in NAcC and action initiation [22], our hypothesis was that D1Rs in NAcC would be a critical locus for this. Therefore, we examined the effects of intra-NAcC infusions of either the D1R agonist or antagonist (cohort 2). To ensure consistency with the effects we observed in the first cohort, prior to surgery we replicated the systemic D1R agonist experiment and found a comparable pattern of effects on No-Go and Go performance (Supplementary Fig. S2; drug × cohort interactions: all *p* > 0.2).

#### No-Go trials

**Fig. 4:**
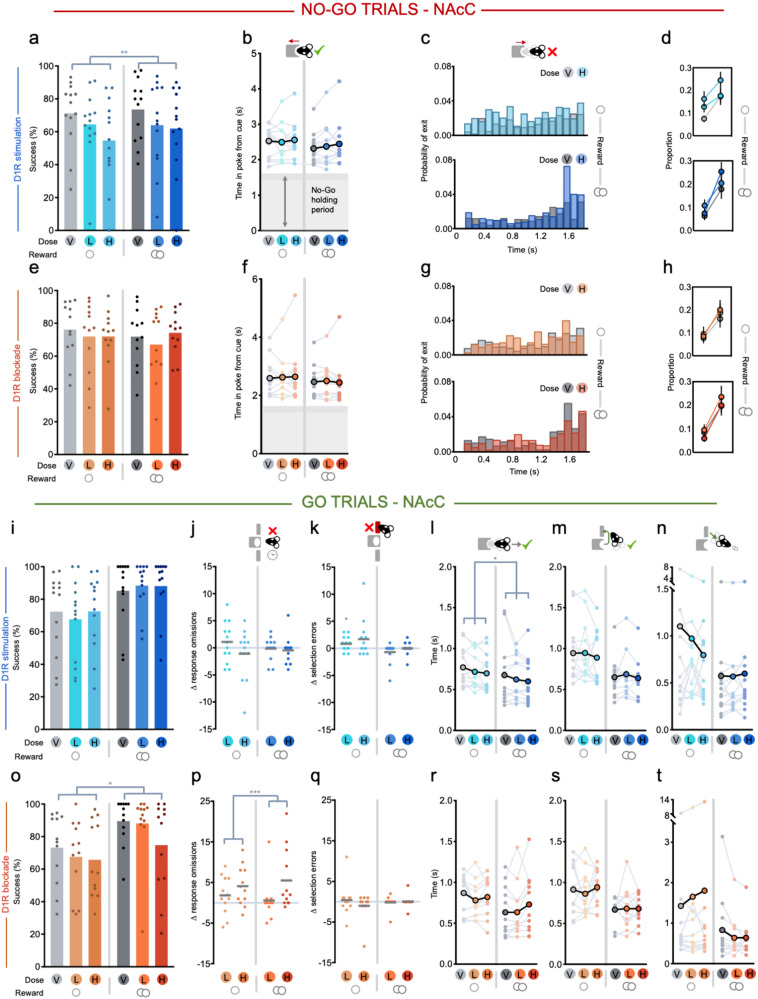
Effects of intra-NAcC D1R stimulation (SKF-81297) or blockade (SCH-23390) in No-Go and Go trials. V vehicle, L low dose, H high dose. Single circle indicates a small reward condition, double circle indicates a large reward condition. **a**, **b** Effects of D1R stimulation split by small (left) and large (right) reward No-Go trials on **a** success rate and **b** time in nosepoke from cue onset in successful trials. Darker shading reflects the jittered No-Go holding period. **c** Mean probability histogram of time in nosepoke in failed small (upper) and large (lower) reward No-Go trials for saline (gray) or high dose (orange and red) manipulations, calculated as a probability over all head exit times. (Pairwise comparisons: early period vehicle vs. low dose: *p* = 0.019, vehicle vs. high dose: *p* = 0.022; late period vehicle vs. high dose: *p* = 0.009, vehicle vs. low dose: n.s., *p* > 0.6). For this analysis, we excluded one animal where on average >50% of the errors occurred in the early No-Go period, which was >3 SD from the group. **d** Mean proportion of times spent in the nosepoke across trials that were early (<800 ms) or late (>800 ms) for small (upper) and large (lower) reward trials. **e**–**h** Same as in (**a**–**d**) but for local D1R blockade. **i**–**n** Effects of local D1R stimulation split by small (left) and large (right) reward Go trials on **i** success rate, **j** response omission errors, **k** lever selection errors, **l** latency to leave the nosepoke after Go cue onset, **m** latency from nosepoke exit to first lever press, **n** and latency from trial completion to entering the food magazine to retrieve the reward. **o**–**t** Same as in (**i**–**n**) but for local D1R blockade. **p < 0.01, *p < 0.05.

Intra-NacC administration of a D1R agonist or antagonist replicated most effects of systemic administration; NAcC D1R stimulation increased premature responses after cue onset on No-Go trials (Fig. 4a; main effect of drug: *F*_(2,24)_ = 8.459, *p* = 0.002) and this was again particularly evident early in the No-Go holding period, although here the highest dose also increased errors in the late period (Fig. 4c, d; main effect of drug: *F*_(2,22)_ = 6.630, *p* = 0.006; drug × period interaction: *F*_(2,22)_ = 3.613, *p* = 0.044). On correctly performed No-Go trials, as before, there were no reliable changes in the speed to exit the nosepoke (Fig. 4b) or to reach the magazine (all *F* < 2.7, *p* > 0.09). Intra-NAcC infusion of the D1R antagonist again had no effect on performance or latencies in No-Go trials, replicating the pattern of results from systemic administration (Fig. 4e–h; all *F* < 1.6, *p* > 0.2).

To investigate what was driving this increase in premature errors on No-Go trials, we used video tracking on a subset of rats for which we were able to perform video analyses (*n* = 6, see Supplementary Methods, Supplementary Fig. S3a, b). Rats were more likely to directly visit the food magazine than either lever, particularly when a large reward was available (Supplementary Fig. S3c–e; main effect of location: *F*_(2,8)_ = 13.448, *p* = 0.003; location × reward interaction: *F*_(2,8)_ = 4.899, *p* = 0.041). Importantly, this response pattern was comparable after intra-NAcC D1R agonist administration (Supplementary Fig. S3c–e; main effect of drug, drug × reward × location interaction, both *F* < 1.6, *p* > 0.25), the only difference being that the drug tended to reduce the likelihood of reaching any target location on small reward trials (drug × reward interaction: *F*_(1,4)_ = 27.495, *p* = 0.006). Therefore, although stimulation of NAcC D1Rs increased the likelihood of premature No-Go responses in the presence of reward-associated cues, this was not driven by a selective change in response strategy toward the levers or food magazine.

#### Go trials

Intra-NAcC administration of the D1R agonist or antagonist had more selective effects than was observed after systemic administration. Unlike systemic administration, stimulation of NAcC D1Rs had no overall effect on the proportion of correct Go responses (Fig. 4i; main effect of or interaction with drug: both *F* < 2.3, *p* > 0.1). It promoted faster action initiation (Fig. 4l; main effect of drug: *F*_(2, 24)_ = 4.046, *p* = 0.031), although, unlike with systemic administration, neither the speed with which animals traveled to the lever or retrieved the reward were affected (Fig. 4m, n; both *F* < 0.9, *p* > 0.4).

Blockade of NAcC D1Rs resulted in a lower success rate in Go trials, mirroring the effect with systemic administration (Fig. 4o; main effect of drug: *F*_(2, 22)_ = 4.559, *p* = 0.022), and this was again caused by a selective increase in response omissions (Fig. 4p; main effect of drug: *F*_(2, 22)_ = 4.542, *p* = 0.022; lever selection errors both *F* < 1.9, *p* > 0.18; Fig. 4q). However, whereas systemic D1R blockade had significantly slowed distal latencies, here, surprisingly, intra-NAcC administration of the D1R antagonist had no effect on any latencies (Fig. 4r–t; time in poke from cue, travel time, and reward retrieval: no main effects or interactions with drug, all *p* > 0.09).

### Focused responding on Go trials is shaped by reward and is mediated by NAcC D1Rs

To understand this surprising disconnection between the observed increase in response omissions on incorrect Go trials after intra-NAcC D1R blockade and the absence of an effect on response latencies on correctly performed Go trials (Fig. 4r–t), we performed finer-grained analyses of Go trial performance.

**Fig. 5:**
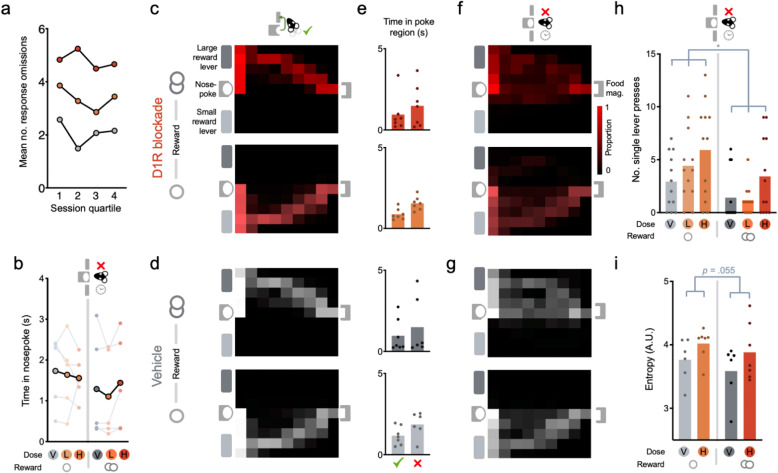
Effects of intra-NAcC D1R blockade (SCH-23390) in Response Omission Go trials. **a** Mean number of response omission errors made across rats across sessions when each session is split into quartiles. **b** Mean time in nosepoke from cue onset in response omission trials. **e** Mean time in the area of the nosepoke (see Methods: Video analyses). **c**, **d**, **f**, **g** Mean probability density across rats in small (lower) or large (upper) reward Go trials, when **c** correct on the high dose of the intra-NAcC D1 antagonist, **d** correct on vehicle, **f** in response omission trials on the high dose of the intra-NAcC D1 antagonist, and **g** in response omission trials on vehicle. **h** Total number of single lever presses in response omission trials. **i** Average entropy of animals in response omission trials. Data displayed for all animals for which we had tracking, but the statistical analysis was restricted to *n* = 5 for which we had reliable tracking in both drug and vehicle sessions.

First, we investigated whether this dissociation could be caused by the intra-NAcC D1R antagonist having a cumulative effect on the ability of rewards to maintain arousal within a session. We reasoned that this would manifest as the correct responses with normal response latencies predominating early in the session and response omissions clustering later in the session. In fact, however, elevated error rates were equally distributed across the session (Fig. 5a; main effect of drug: *F*_(2, 22)_ = 4.609, *p* = 0.021; no main effect of quartile or interaction, both *F* < 0.7, *p* > 0.5). Moreover, there was no evidence that errors were influenced by recent reward (Supplementary Fig. S4a; no main effect of drug: Previous Reward *F* < 0.3, *p* > 0.8) or trial history (Supplementary Fig. S4b: no main effect of drug: Previous Go: *F* < 2.1, *p* > 0.14).

Next, we examined response variables and within-trial trajectories using video tracking on a subset of rats after intra-NAcC administration of either vehicle or the high dose of the D1R antagonist (*n* = 5–7, see Supplementary Methods). We assessed whether the drug caused rats to be more likely either to: (1) “opt out” (i.e., remain near the start port); (2) make the “wrong response” (head to the wrong lever or food magazine); or (3) be “unfocused”, where the appropriate action is taken, but with less vigor and accuracy, thereby resulting in the rat failing to meet the response requirement of the trial.

While animals were slower to initiate actions on omission trials compared to correct Go trials, importantly this was no different with or without intra-NAcC D1R blockade (Fig. 5b; main effect of outcome: *F*_(1,4)_ = 11.816, *p* = 0.026; no main effect of drug or interaction with outcome or reward, all *F* < 1.5, *p* > 0.2; small reward trials only, main effect of outcome: *F*_(1,9)_ = 13.328, *p* = 0.005; no main effect of drug or interaction with outcome, all *F* < 0.9, *p* > 0.4). Similarly, time spent in a defined area near the nosepoke after erroneous head exits in Go trials was unchanged by the intra-NAcC D1R antagonist, suggesting that rats were not “opting out” (Fig. 5e; no main effect of drug or interaction, both *F* < 1.0, *p* > 0.3).

In fact, during the 5-s cue presentation on these omission trials, rats would often perform comparable sequences of actions as on correct Go trials—moving toward the cued lever and even subsequently heading to the food magazine (Fig. 5c–f). Strikingly this pattern was equivalent whether they had been administered the D1R antagonist or vehicle, despite the overall propensity of rats to make omission errors being increased with the antagonist. Specifically, the proportion of omission trials in which rats first visited the region of the correct lever was significantly higher in comparison to first visiting the incorrect lever, but this was unaltered by the drug (average proportion of correct lever responses: vehicle small reward: 0.72 ± 0.11, large reward: 0.75 ± 0.14; SCH small reward: 0.65 ± 0.09, large reward: 0.65 ± 0.15, mean ± SEM; main effect of outcome: *F*_(1,4)_ = 100.791, *p* = 0.001; no main effect of drug, reward, or interactions, all *F* < 0.5, *p* > 0.4) and the cumulative probability of visiting the area near the correct lever when on drug did not significantly differ from vehicle (no main effect of drug or interaction, both *F* < 0.4, *p* > 0.5). There was also no difference due to drug in how likely the rats were to visit the correct lever and then go on complete the trajectory by visiting the magazine (no main effect or interaction with drug, both *F* < 0.5, *p* > 0.5; vehicle small reward: 0.42 ± 0.11, large reward: 0.53 ± 0.21; SCH small reward: 0.39 ± 0.14, large reward: 0.42 ± 0.15). In addition, trajectory lengths during the 5-s cue window were comparable between error and correct trials on or off drug (no main effect of drug or interaction, both *F* < 1.2, *p* > 0.3).

Yet importantly, although trajectories on omission trials contained many features common with correctly performed Go trials, responding on omissions nonetheless lacked equivalent focus and precision. After the intra-NAcC D1R antagonist, rats were more likely to make a single response on the correct lever rather than the two required for the trial to be successful (Fig. 5h; main effect of drug: *F*_(2,22)_ = 5.571, *p* = 0.011). Moreover, the entropy, or noisiness, of the animals’ trajectories in omission trials on and off drug showed a strong trend for entropy to be increased by the NAcC D1R blockade (Fig. 5i; main effect of drug: *F*_(1,4)_ = 7.201, *p* = 0.055). This appeared selective to Go trial sequences as entropy of movement to the magazine on correct No-Go trials was unaltered (Supplementary Fig. S5a; no main effect of drug or interaction: *F* < 0.7, *p* > 0.4). Moreover, there was no evidence of consistent alterations in responding outside of cue-driven Go responses as next trial re-engagement latencies—which were not externally cued—were not reliably altered by the drug (Supplementary Fig. S5b; no main effect of drug: *F* < 2.2, *p* > 0.13). Together this suggests that the promise of reward, signaled by cues, facilitates animals to engage in focused reward-seeking sequences through NAcC D1Rs and that blockade of these signals reduces the likelihood of animals transitioning to this focused reward-seeking state.

## Discussion

Dopamine transmission is a key component mediating the influence of reward predictions on behavior, yet its precise role in cue-driven behavioral control has remained contentious [4, 41–44]. Here we used a factorial design, which separately manipulated reward size and the behavioral requirements to gain that reward, to investigate the role of dopamine transmission at D1Rs in regulating this relationship. Stimulation, but not blockade, of D1Rs across the whole brain or locally in the NAcC consistently disrupted No-Go performance, potentiating premature action initiation that clustered soon after cue presentation. The most prominent effect of D1R blockade, by contrast, was to increase response omissions on Go trials. While this manifested as a selective reduction in the vigor of distal actions in the response sequence when D1Rs were blocked globally, after intra-NAcC blockade these metrics were unaffected. Instead, disruption of NAcC D1Rs increased the probability that Go trial performance was in an “unfocused” state, characterized, both on and off drug, as a reduction in the precision of responding even though the appropriate action sequence was often executed.

The prospect of reward can positively shape both the speed and precision of behavior [30, 45–47], and several lines of evidence suggest that dopamine may play a key role in mediating aspects of both processes [24, 26, 29, 30, 48]. As expected, rats’ performance in the current experiment was strongly affected by the reward size on offer. Cues associated with a large future reward reduced action latencies to complete each element of the action sequence. This finding is consistent with the notion that there is a direct link between the vigor of actions—the reciprocal of the time to complete an action sequence [47]—and the net gain from obtaining the potential reward [24, 49, 50]. However, there was an asymmetric influence on response accuracy; the prospect of a large reward improved Go trial accuracy, but had no reliable effect on successful No-Go trial completion. This could be caused by reward having distinct influences on separable processes during No-Go trials, boosting not only instrumental precision but also a Pavlovian draw toward rewarded locations, which here is maladaptive [45, 51]. Indeed, when animals exited the nosepoke prematurely on No-Go trials, we found that they tended to approach the food magazine, particularly when a large reward was on offer (Supplementary Fig. S3).

While the presentation of cues associated with future reward can rapidly increase dopamine levels in terminal regions in relation to the value of available reward [15–17, 52], we and others have found that release patterns are suppressed until a reward-seeking action is initiated [21, 22]. Pronounced changes in dopamine can increase the excitability of D1-expressing MSNs [5, 35, 39]. Here, we found that pharmacological stimulation of D1Rs rapidly promoted actions to be initiated, typically speeding action initiation on Go trials but also consistently increasing inappropriate No-Go responses. These premature actions were most evident early in the No-Go holding period just after cue presentation. Given that the prospect of high reward *reduced* early No-Go errors in baseline testing, this implies that D1R stimulation did not increase the state value but instead promoted action initiation. This aligns with the idea that dopamine influences the likelihood of engaging with “work” [29] while specifying that work does not just mean cognitive control, but specifically the activation of motor programs to pursue a rewarding opportunity [41]. It is possible that this is due to the longer timescales over which pharmacological manipulations act, and that a more temporally precise manipulation of activity at these receptors would instead alter state value. Future studies that employ techniques with greater temporal specificity than is achievable using pharmacology will be helpful to test these ideas.

The lack of an increase in head exits during the pre-cue period suggests that cue presentation was critical to elicit the behavioral response. This contrasts with the effects of intra-NAcC administration of amphetamine, which caused increases in both early and late impulsive actions on No-Go trials and in aborted trials during the pre-cue period [33]. Therefore, while these findings are broadly consistent with studies implicating hyperdopaminergic states with an increased likelihood of motor or “waiting” impulsivity [10, 13], our task here allows us to pinpoint the role of D1R transmission, particularly in NAcC, in facilitating cues signaling reward opportunities to promote transitions to action. Nonetheless, as intra-NAcC D1R blockade had no effect on No-Go performance, it is clear that D1R activation is not *necessary* for actions to be executed.

Cue-evoked excitation of D1-expressing MSNs has previously been closely tied to the latency to initiate reward-seeking behavior [3, 5]. Of particular relevance, in one recent study, du Hoffmann and Nicola showed that intra-NAcC administration of D1 agonists increased cue-driven reward seeking in a state of satiety [53], which separate work has shown to attenuate dopamine release to reward-associated cues [17, 54]. While systemic manipulation of D1Rs affected response latencies during several elements of the action sequence, the role of NAcC D1Rs was instead specific to action initiation. One possibility is that regulation of ongoing movement vigor, particularly in the service of gaining response-contingent rewards, relies on D1Rs in the dorsal striatum [55–57]. Notably, both optogenetic inhibition and stimulation of substantia nigra pars compacta dopamine cells or D1-expressing MSNs have been shown to disrupt ongoing movements [58, 59], which parallels the effect observed here that systemic administration of not just the D1R antagonist but also the D1R agonist slowed travel to the lever. The latter manipulation also caused a small but reliable increase in incorrect lever presses on Go trials, and both effects may reflect competition between different potential reward-associated instrumental responses in dorsal striatum [59].

Given the importance of NAcC D1Rs in regulating decisions to act and also in modulating arousal [60], it might initially seem obvious that intra-NAcC D1R blockade would also cause an increase in the proportion of response omissions on Go trials. However, two aspects make this result more surprising. First, a number of elegant experiments have shown that NAcC dopamine transmission is particularly important for flexible or taxic responses—in other words, when needing to take a novel path to gain reward [3]—yet here the start and goal locations are fixed across trials. Second, this increase in omissions occurred alongside an absence of an effect on any latency measures on correctly performed trials. When considered alongside the lack of any change in No-Go performance, these effects appear hard to account for by a simple sustained change in arousal. Although manipulations of mesolimbic dopamine have been shown to influence wakefulness [61], there is no evidence for a relationship between dopamine activity and fluctuations in pupil size, a standard measure of autonomic arousal, during cost-benefit decision making [62]. Similarly, it seems unlikely the D1R antagonist reduced the efficacy of rewards to maintain behavioral engagement [63], as omission error rates were comparable from the start to the end of the session. There was also no evidence that the rats were simply disorganized or disengaged during omissions after D1R administration; analysis of the patterns of responding in a subset of animals showed that they performed many of the same action sequence components on these trials as observed on correctly performed Go trials.

Instead, what characterized performance on response omissions was a marked reduction in the *precision* in the execution of the response sequence. This did not primarily affect the overall direction of the response, similar to previous reports [9, 64] but instead involved slower initiation, less focused responses toward the correct lever (i.e., increased entropy of response trajectories), and increased likelihood of only making one of the two required lever presses. Crucially, this unfocused state had not emerged de novo with the administration of the intra-NAcC D1R antagonist, but instead reflected a potentiation of an analogous response pattern observed off drug. Response omissions in baseline sessions most commonly occurred on small reward trials, which generate an initial dip in NAcC dopamine [22]. Nonetheless, it is important to note that stimulation of NAcC D1Rs did not concomitantly increase the success rate on Go Small reward trials. Therefore, whilst D1R transmission is necessary to facilitate transitions to focused reward seeking, it is not sufficient in the absence of other inputs. Moreover, both high and low reward trials appeared comparably affected. As such, it may be that this observed reduction in the ability of reward-associated cues to promote focused reward-seeking actions under NAcC D1R blockade could be considered a refinement of the broader term of “arousal” [60], incorporating ideas about stimulus salience and from activational theories of mesolimbic dopamine [4, 9, 65, 66] (see also [67]).

Considering the tight relationship between rapid changes in dopamine and the value of an anticipated future reward [15–17, 52] and exertion of effort [9], it might have been expected that the pharmacological manipulations would disrupt the influence of reward size over response vigor. Instead, latencies remained consistently faster on large reward trials and any effects of drug were of comparable size irrespective of the reward on offer. One possibility is that the general motivational influence of reward might be mediated through D1- and D2Rs and therefore both might need to be targeted to disrupt the effect of reward expectation on action invigoration. It has been shown that blockade of either D1 or D2 receptors in NAcC similarly attenuates excitation evoked by reward-associated cues [5]. In addition, it may be that in highly-trained animals performing a task with stable cue-action-reward associations, invigoration of the stored action sequence becomes less reliant on the magnitude of dopamine release. Finally, it may well be that NAcC is not the sole locus for these effects. For example, ventral pallidum, which receives direct input from prefrontal regions as well as NAcC, also responds strongly to reward-predictive cues with similar or even faster latencies than NAcC neurons, and promotes instrumental action [68, 69].

Together, this demonstrates that an appropriate balance of activity at NAcC D1Rs is critical to regulate proficient and focused reward seeking. Activation of NAcC D1Rs, such as will occur via endogenous dopamine release in response to cues signaling an improved reward opportunity, plays a key role in promoting rapid transitions to action. While this is beneficial to promote the initiation of a focused reward-seeking response sequence, it can also be problematic in situations where response restraint is required. In turn, however, in the absence of D1R activity, animals are more likely to act in an unfocused state, causing failures to successfully complete each element of a required reward-seeking sequence. This may be relevant for understanding the actions of therapeutic doses of stimulant drugs such as amphetamine, which can potentiate evoked NAcC dopamine and increase sustained attention [70, 71].

## Supplementary information

Supplementary information

## Data Availability

All datasets are available from the corresponding author on reasonable request.
