# Correction to: Nucleus accumbens D1-receptors regulate and focus transitions to reward-seeking action

**DOI:** 10.1038/s41386-022-01343-z

**Published:** 2022-05-24

**Authors:** Laura L. Grima, Marios C. Panayi, Oliver Härmson, Emilie C. J. Syed, Sanjay G. Manohar, Masud Husain, Mark E. Walton

**Affiliations:** 1 Department of Experimental Psychology, University of Oxford, Oxford, UK; 2 Medical Research Council Brain Network Dynamics Unit, University of Oxford, Oxford, UK; 3 Nuffield Department of Clinical Neurosciences, University of Oxford, Oxford, UK; 4 Wellcome Centre for Integrative Neuroimaging, University of Oxford, Oxford, UK; 5 Present Address: Janelia Research Campus, Howard Hughes Medical Institute, Ashburn, VA USA; 6 Present Address: National Institute on Drug Abuse, Biomedical Research Center, Baltimore, MD USA

**Keywords:** Reward, Motivation

Correction to: *Neuropsychopharmacology* 10.1038/s41386-022-01312-6, published online 27 April 2022

In the sentence beginning “Behavior on Go and No-Go...” and onward, the value] “p” should have read “F”. The original article has been corrected.

