# Supplementary material for: Nucleus accumbens D1-receptors regulate and focus transitions to reward-seeking action
Source: Neuropsychopharmacology. 2022 Apr 27;47(9):1721–31. doi: 10.1038/s41386-022-01312-6 (PMC9283443; doi:10.1038/s41386-022-01312-6)
Supplement: Supplementary file 1 — Supplementary information [file 41386_2022_1312_MOESM1_ESM.pdf]

Supplemental methods

Subjects

Rats were aged between 8 and 12 weeks at the beginning of training. Two cohorts of rats were used; Cohort 1 consisted of 11 rats previously implanted for use in an FCV study (1), and Cohort 2 consisted of 14 naïve rats. Note that the rats in Cohort 1 also received the D1R antagonist systemically, but due to issues with the drug preparation, the incorrect doses were administered and the dataset was excluded. No statistical methods were used to pre-determine sample sizes, but sample sizes are comparable to those reported in previous publications. All animals were maintained on a twelve-hour light/dark cycle. All testing was carried out during the light phase, and during training and testing periods animals were food restricted to 85-90% of their free-feeding weight. Water was provided ad libitum in the home cage.

Experiment	Cohort	Original n	Results n	Reason for Exclusions
Systemic D1 agonist	1	11	11	–
Systemic D1 antagonist	1	11	–	Incorrect dosing of drug
Systemic D2 agonist	1	11	9	Computer failure (n=2)
Systemic D2 antagonist	1	11	10	Computer failure (n=1)
Systemic D1 agonist (replication)	2	14	14	–
Systemic D1 antagonist	2	14	13	Performance (n=1)
Local D1 agonist	2	14	13	Misplaced cannulae (n = 1)
Local D1 antagonist	2	14	12	Misplaced cannulae (n=1),<br>Performance (n=1)

Table S1. Experimental details, sample sizes and reasons for exclusions. In the case of 'performance' exclusions, subjects were excluded if they completed <20% of trials in a session (n = 2 across all experiments).

Apparatus and behavioral training

Animals were trained on an operant Go/No-Go task (Fig. 1a, b) in which, after initiating a trial by making a nosepoke, auditory cues instructed them either to make (Go) or withhold (No-Go) action in order to gain either a small or large reward. Experiments were conducted using MED-PC behavioral chambers fitted on

one wall with two retractable levers 9.5cm on either side of a central nosepoke, and a food magazine on the opposite wall into which 45mg sucrose pellets (Test Diet, Sandown Scientific, UK) were dispensed. Both the nosepoke and food magazine were fitted with infrared beams for entry detection. Each chamber was also fitted with a speaker for delivering the 4 auditory stimuli (~70dB tone, buzz, white noise, or clicker) and a house light.

After magazine training, animals were first trained on the No-Go trial type. On these trials, the rat was required to remain in the nosepoke for the required period. The No-Go duration was incrementally increased across training sessions on reaching the behavioral criterion (≥ 60% success rate) up to a jittered pre-cue period of 0.3-0.7s and a maximum cued hold period of 1.5-1.7s. A 0.1s ‘buffer’ period was also introduced to distinguish between genuine nosepoke exists and small shifts in posture that may have inactivated the poke detector. Successful trials were rewarded with either one (small reward) or two (large reward) sucrose pellets, as cued by the auditory stimulus.

After reaching criterion for both No-Go trial types, animals were next trained on the Go trial type. Mirroring the No-Go trials, correct choice of one lever (either left or right, side counterbalanced across animals) was rewarded with one pellet (small reward), whilst the other was rewarded with two pellets (large reward), again cued by the auditory stimulus. After again reaching criterion, No-Go trials were interleaved with Go trials to give the full task, in which animals experienced all four trial types pseudorandomly.

Behavior – full task

Cues were counterbalanced across animals. In No-Go trials, the cue sounded until the end of the hold period or, if the rats exited the nosepoke prematurely, until the time of exiting the poke. In Go trials, the cue sounded until animals pressed the correct lever twice, or until they pressed the wrong lever, or for a maximum of 5s if they failed to press any lever (response omissions). In the full Go/No-Go task (Fig. 1a, b),

animals initiated a trial by entering and remaining in the nosepoke for the pre-cue hold period (jittered in 0.1s increments between 0.3-0.7s). This led to presentation of one of 4 auditory cues indicating the action required (Go or No-Go) and the size of the reward on offer (small or large). On correct trials, rewards were delivered 1s after successful completion of a trial. After reward delivery a 5s inter-trial interval (ITI) commenced. No cue indicated the end of the ITI and animals were free to initiate the next trial after this time. Both failed Go and No-Go trials resulted in the house light illuminating for a 5s time-out period 1s after the error before turning off and the 5s ITI commencing. The session ended after animals had either gained 100 rewards or after 60 minutes. Key task performance and latency measures are outlined in Table S2.

Performance measures					
Trial type	***Success***	***Success – other***	***Error type***	***Error – other***	***Other***
Go	Correct trials [%]	Additional lever presses [per trial]	i. Response omission<br>ii. Wrong lever [%]	N/A	Aborted [per trial]<br>*Nose-poke exit during pre-cue period*
No-Go	Correct trials [%]	N/A	Premature exit (early / late) [%]	Invalid lever press [%]<br>*Lever press after premature exit*	Aborted [per trial]<br>*Nose-poke exit during pre-cue period*
**Latency measures (success)**					
Go	Time in poke from cue [s]<br>*Cue onset to nose-poke exit*	Travel time [s]<br>*Nose-poke exit to first lever press*	Inter-press [s]<br>*First to second lever press*	Reward retrieval [s]<br>*Second lever press to magazine entry*	Re-engagement [s]<br>*First nose-poke after success or 1s after failure*
No-Go	Time in poke from cue [s]<br>*Cue onset to nose-poke exit*	N/A	N/A	Reward retrieval [s]<br>*Nose poke exit to magazine entry*	Re-engagement [s]<br>*First nose-poke after success or 1s after failure*

Table S2. Summary of key performance and latency measures in the Go/No-Go task.

Analysis of behavior

All behavioral measures were calculated on a session-by-session basis. Data was extracted and analysed using MATLAB R2018a and IBM SPSS Statistics 24. Significant interactions were explored by analysis of the simple effects and are reported in the appropriate figure legends or tables. Performance in this study for each trial type was expressed as percentage success over all attempted trials within a session. In analysing baseline behavior for the results shown in Figure 2, data was pooled across all vehicle sessions from the systemic and local experiments where all doses of the drug were administered. Randomisation or blinding was not used during analysis.

Video tracking

We used the DeepLabCut toolbox (2) in order to perform online pose estimation from videos acquired during behavioral sessions. Videos were captured at 25 fps. Nodes included the rats' nose, ears, head, body, legs, and tail, as well as key features of the operant chambers – the nosepoke, left and right levers, and left and right corners of the food magazine. Two separate models with matching parameters were trained to account for differences in box orientation. For each video, 25 randomly selected frames were manually labelled before the network was trained and tested over 1030000 iterations, resulting in an average tracking error of < 5 pixels. After training, only frames with co-ordinates that had a likelihood of 1 were included and any missed frames were interpolated across. These co-ordinates were aligned with MED-PC behavior data by identifying when animals made errors in the task as indicated by a sharp increase in average luminance values from gresyscale converted video frames due to the houselight being turned on. Reasons for excluding sessions included a failure to align tracking with MED-PC behavior, poor visibility of operant chamber features, and sessions in which a majority of frames required interpolation. X and y co-

ordinates from the tracked nose marker were used for all analyses. Any negative values in the y axis were due to the rat having its nose in the nosepoke and were therefore converted to 0.

We normalised the values of operant chamber parts on a per session basis by subtracting the median co-ordinates of the nosepoke from the values of the tracked parts. For all behavioral metrics relating to analysis of rat location in the chamber relative to chamber components (aside from the calculation of entropy – explained below), we first divided the range of box space co-ordinates into a 3 x 3 grid, allowing for two squares of the grid to be labelled as lever squares, one as a nosepoke square, and one as a magazine square. For each frame, if a given co-ordinate was within the boundaries of a square it was scored as '1', or '0' otherwise. This was averaged across to give a mean probability density function across trials for each rat, and then averaged across rats. To calculate the time spent in the area of the nosepoke we measured the latency from the beginning of the trial to when the animals were detected to have left this square based on the tracked nose co-ordinates being outside of the boundary of the nosepoke square. To calculate the proportion of trials on which animals completed a particular sequence of actions, e.g. moving from the correct lever to the food magazine, we again used this binary measure of whether the tracked co-ordinates had entered within the specified squares but in addition specified a required order of visitation for the trial to be counted as such. Trajectory lengths were calculated by finding the Euclidean distance between frames and normalized by the median distance between the lever markers before being summed and averaged. For the calculation of entropy, we divided the range of box space co-ordinates into an 18 x 18 grid in order to increase the granularity of the analysis. We then normalized the probability values of each location, L, in the grid on a per session basis before calculating entropy using the Shannon equation (3) for each session as: $E = -\sum p_L \, log_2 p_L$. For the colorplot visualizations in Fig. 6 and Fig. 8, the box boundaries were reduced by 50 pixels along the x axis as very few tracking points reached those co-ordinates.

Pharmacological challenges

All compounds were acquired from Tocris Bioscience. Doses were calculated as the salt. All drugs were dissolved in 0.9% sterile saline, made in batch, aliquoted, and frozen at -20°C. Individual aliquots were defrosted for use on testing days. 0.9% sterile saline was administered in control sessions. In all experiments, doses were applied in a counterbalanced Latin square approach, although blinding was not used when making up or applying agents.

For eticlopride, we based our doses on published studies that show effects of eticlopride on operant reward-guided decision-making tasks (e.g. (4–6)).

Compound	Locus / direction of effect	Route of administration	Doses (mg/kg or µg/µl)
SKF-81297 hydrobromide	D1R agonist	Systemic (i.p. injection)	0.3, 1.0
		Local (intracranial infusion)	0.4, 4.0
SCH-23390 hydrochloride	D1R antagonist	Systemic (i.p. injection)	0.005, 0.01
		Local (intracranial infusion)	0.2, 2.0
Quinpirole hydrochloride	D2R agonist	Systemic (i.p. injection)	0.0125, 0.0375
Eticlopride hydrochloride	D2R antagonist	Systemic (i.p. injection)	0.01, 0.03

Table S3. Summary and details of pharmacological compounds used across experiments.

Surgical procedure

Bilateral guide cannulae were implanted 1.5mm above the target site of the NAcC, at co-ordinates of AP relative to bregma: +1.4mm, ML: ±1.7mm, DV: -6.0mm from surface of skull relative to bregma. To implant these cannulae, rats were anaesthetised using inhaled isoflurane (4% vol/vol in O_2 induction and 1.5% for maintenance delivered via facemask) and administered buprenorphine (Vetergesic, 0.03 mg/kg, s.c.), meloxicam (Metacam, 2mg/kg, s.c.) and 3ml glucosaline (Aquapharm). Body temperature was maintained at 37±0.5°C by a homeothermic heating blanket. Once animals were secured in a stereotaxic frame (Kopf Instruments) and their scalp shaved and cleaned with dilute hibiscrub and 70% alcohol, a local anaesthetic (bupivacaine, 2mg/kg) was administered to the incision site. Eye gel (Lacri-Lube, Allergan) was applied to the eyes for protection. The skull was then exposed and the skull was levelled based on measurements of Bregma and Lambda. Six holes were drilled: two for implantation of bilateral guide cannulae (Plastics One, UK) and four for anchoring screws (Precision Technology Supplies). Guide cannulae were then lowered. The cannulae consisted of an 8mm plastic pedestal holding two 26-gauge metal tubes with a center-to-center distance of 3.4mm and a length of 7.5mm. Dental acrylic (Associated Dental Products Ltd.) was then applied to secure the cannulae to the skull and screws. After surgery, bilateral dummy cannulae were inserted to ensure patency, and a dustcap was secured to the pedestal. Animals were again administered buprenorphine, meloxicam, and glucosaline post-surgery, and meloxicam was given up to a further three days post-surgery. Animals were group housed after initial recovery and began re-training once they were fully recovered, on average two weeks after surgery.

Systemic administration procedure

Drugs were injected intraperitoneally (i.p.) at a volume of 1 ml/kg bodyweight. All drugs were injected 10 minutes before the behavioural session aside from the D1R antagonist, which was administered 20 minutes before the start of the session. Drug administration sessions were separated by at least one treatment-

free training day to ensure a return to baseline performance and complete washout of the drug, with criteria of ≥ 60% successful trials across all trial types, and session completion within 60 minutes. If these criteria were not met, animals continued with treatment-free training days until performance reach criteria, at which point the next testing day commenced, though in practice animals' performance almost always reached criteria at the first training day.

Local administration procedure

A mock infusion was carried out one day prior to the first experimental session to reduce potential tissue damage-related confounds. This involved insertion of the injectors back-filled with saline without infusion of any substance. The following day two 10μl glass Hamilton syringes were back-filled with 0.9% sterile saline and placed in an infusion pump (Cole-Parmer). Double connector assembly tubing (Plastics One, UK) was cleaned with ethanol and thoroughly dried with air, then filled with saline before being attached to the Hamilton syringes. 33-gauge 9mm bilateral injectors (Plastics One, UK) that had been cleaned by sonication for one hour in 70% ethanol were then attached to the connector assembly. A small air bubble separated the saline from drug. The injectors were checked for blockage before the rats were gently restrained, their dummy cannulae removed, and injectors inserted. During infusion, 0.5μl of solution was injected per hemisphere at 0.25μl/minute. Injectors were left in place for a further two minutes after infusion and then removed. The dummy cannulae and dustcap were replaced and rats were returned to their homecage for 10 minutes before beginning the task.

Histology

At the end of data collection, cannulated animals were deeply anaesthetised with sodium pentobarbitone (200mg/kg, i.p. injection) and transcardially perfused with 0.9% saline followed by a 10% formalin solution

(vol/vol). Brains were kept in 10% formalin solution until being sectioned. Brains were sectioned into 60μm-thick coronal sections by vibratome (Leica). The sections were stained with cresyl violet (Sigma Aldrich) before being mounted in DePeX mounting medium onto 1.5% gelatin-coated slides and enclosed with coverslips to confirm cannulae placements (Fig. S6).

SUPPLEMENTAL REFERENCES

1. Syed ECJ, Grima LL, Magill PJ, Bogacz R, Brown P, Walton ME (2016): Action initiation shapes mesolimbic dopamine encoding of future rewards. *Nat Neurosci* 19: 34–36.
2. Mathis A, Mamidanna P, Cury KM, Abe T, Murthy VN, Mathis MW, Bethge M (2018): DeepLabCut: markerless pose estimation of user-defined body parts with deep learning. *Nat Neurosci* 21: 1281–1289.
3. Shannon CE (1948): A mathematical theory of communication. *Bell Syst Tech J* 27: 623–656.
4. St. Onge JR, Floresco SB (2009): Dopaminergic modulation of risk-based decision making. *Neuropsychopharmacology* 34: 681–697.
5. Cocker PJ, Dinelle K, Kornelson R, Sossi V, Winstanley CA (2012): Irrational choice under uncertainty correlates with lower striatal D2/3 receptor binding in rats. *J Neurosci* 32: 15450–15457.
6. Rogers RD, Wong A, McKinnon C, Winstanley CA (2013): Systemic administration of 8-OH-DPAT and eticlopride, but not SCH23390, alters loss-chasing behavior in the rat. *Neuropsychopharmacology* 38: 1094–1104.

Supplemental results

Supplementary text

Supp. Text 1: Global D2R stimulation modulates action vigor

To understand how specific the observed systemic effects were to D1R manipulation, we also investigated the effect of systemic administration of either a D2R agonist or antagonist (both cohort 1). The D2R agonist increased the proportion of premature responses during the pre-cue period (main effect of drug: $F_{(2,16)}$ = 3.652, p = .049), but neither the agonist nor antagonist had any effect on No-Go success, time spent in the nosepoke on successful No-Go trials, nor the distribution of early and late No-Go errors (all p > .1, data not shown).

However, the D2R agonist did markedly slow reward retrieval latencies in No-Go trials (main effect of drug: $F_{(2,14)}$ = 23.044, p < .001) and this effect was paralleled by an overall slowing of movements in Go trials. It strongly decreased success rates (Supp. Fig. 1a; main effect of drug: $F_{(2,16)}$ = 45.299, p < .001) – mainly due to an increase in response omissions (Supp. Fig. 1b; main effect of drug: $F_{(2,16)}$ = 58.576, p < .001; lever selection errors, Supp. Fig. 1c; all p > .06) – and also slowed all latencies, including time in poke from cue (at the highest dose), travel time, and reward retrieval (Supp. Fig. 1d-f; all F > 7, p < .004; also reward retrieval: drug x reward interaction: $F_{(2,16)}$ = 7.962, p = .005). In contrast, the D2R antagonist had no reliable effect on any measure of either No-Go or Go performance or response time (Supp. Fig. 1g-l; all p > .08). Together, these results show that stimulating D2Rs reduces the vigor of all actions – both in Go and No-Go trials – an effect distinct to the influence of D1Rs which mainly affected the vigor of actions distal to reward.

Supplementary figure 1

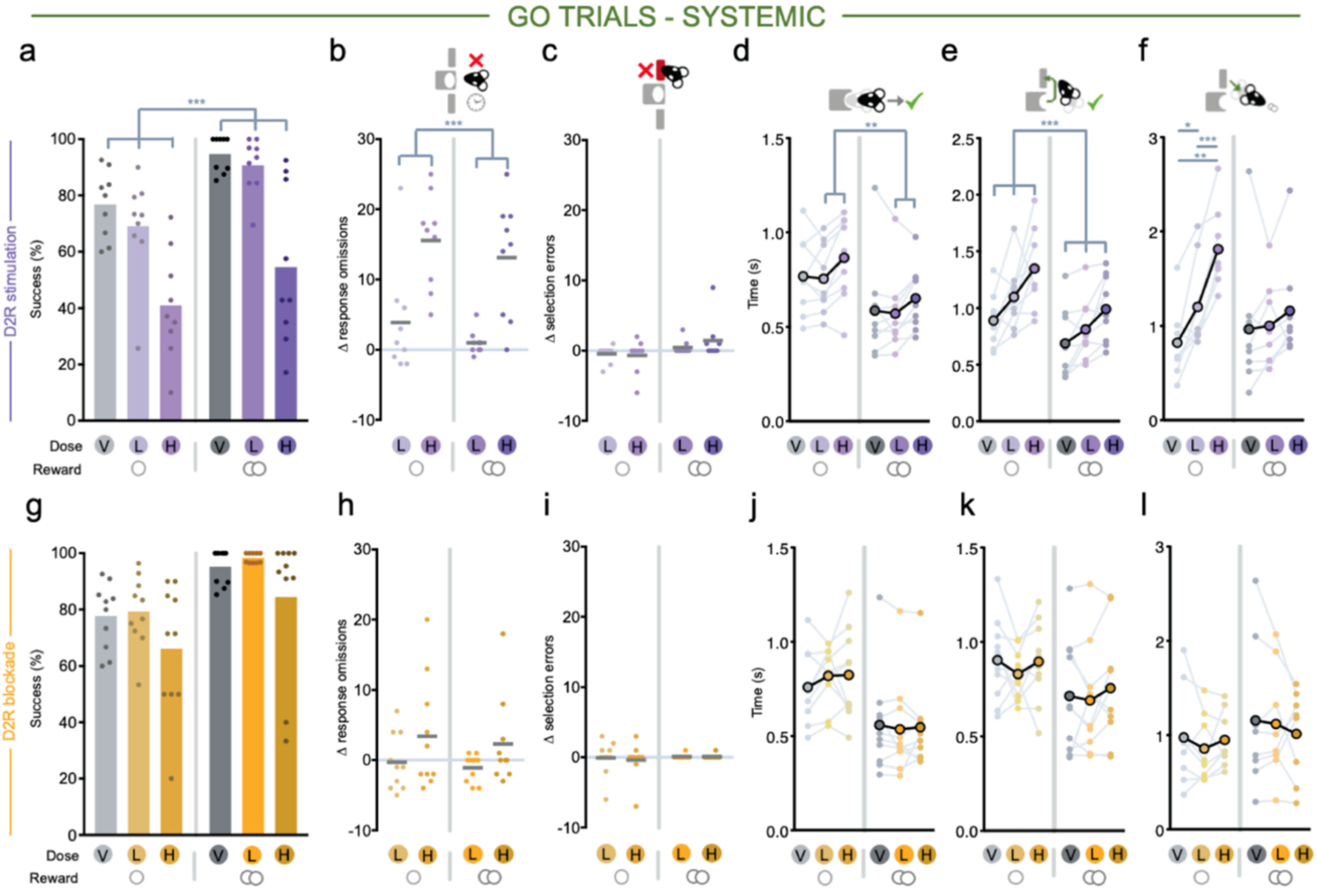

Supp. Fig. 1. Systemic effects of D2R stimulation (quinpirole) or blockade (eticlopride) in Go trials. V = vehicle, L = low dose, H = high dose. Single circle indicates small reward condition, double circle indicates large reward condition. **(a-f)** Effects of D2R stimulation split by small (left) and large (right) reward Go trials on **(a)** success rate, **(b)** response omission errors, **(c)** lever selection errors, **(d)** latency to leave the nosepoke after Go cue onset, **(e)** latency from nosepoke exit to first lever press, **(f)** and latency from trial completion to entering the food magazine to retrieve reward. **(g-l)** Same as in **(a-f)** but for systemic D2R blockade. ****p* < .001, ***p* < .01, **p* < .05

Supplementary figure 2

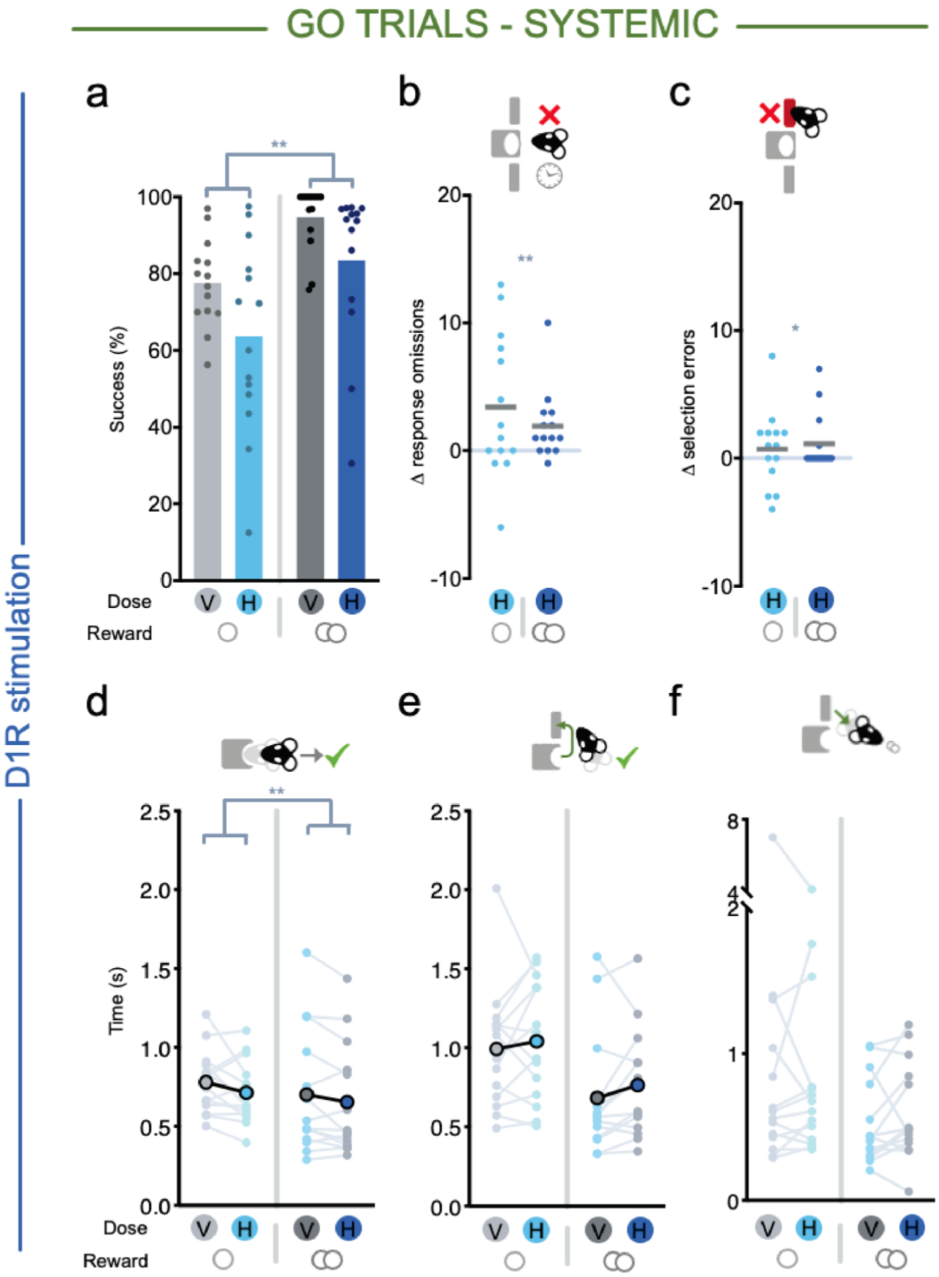

Supp. Fig. 2. Systemic D1R stimulation replication study results. Effect of D1R stimulation on **(a)** success rate (main effect of drug: $F_{(1,13)} = 14.237$, $p = .002$), **(b)** response omission errors (main effect of drug: $F_{(1,13)} = 11.424$, $p = .005$), **(c)** lever selection errors (main effect of drug: $F_{(1,13)} = 4.694$, $p = .049$) **(d)** latency to leave the nosepoke after Go cue onset (main effect of drug: $F_{(1,13)} = 10.895$, $p = .006$), **(e)** latency from nosepoke exit to first lever press (main effect of drug and interaction n.s., $p > .1$), **(f)** and latency from reward delivery to entering the food magazine to retrieve reward (main effect of drug and interaction n.s., $p > .3$). ** $p < .01$, * $p < .05$

Supplementary figure 3

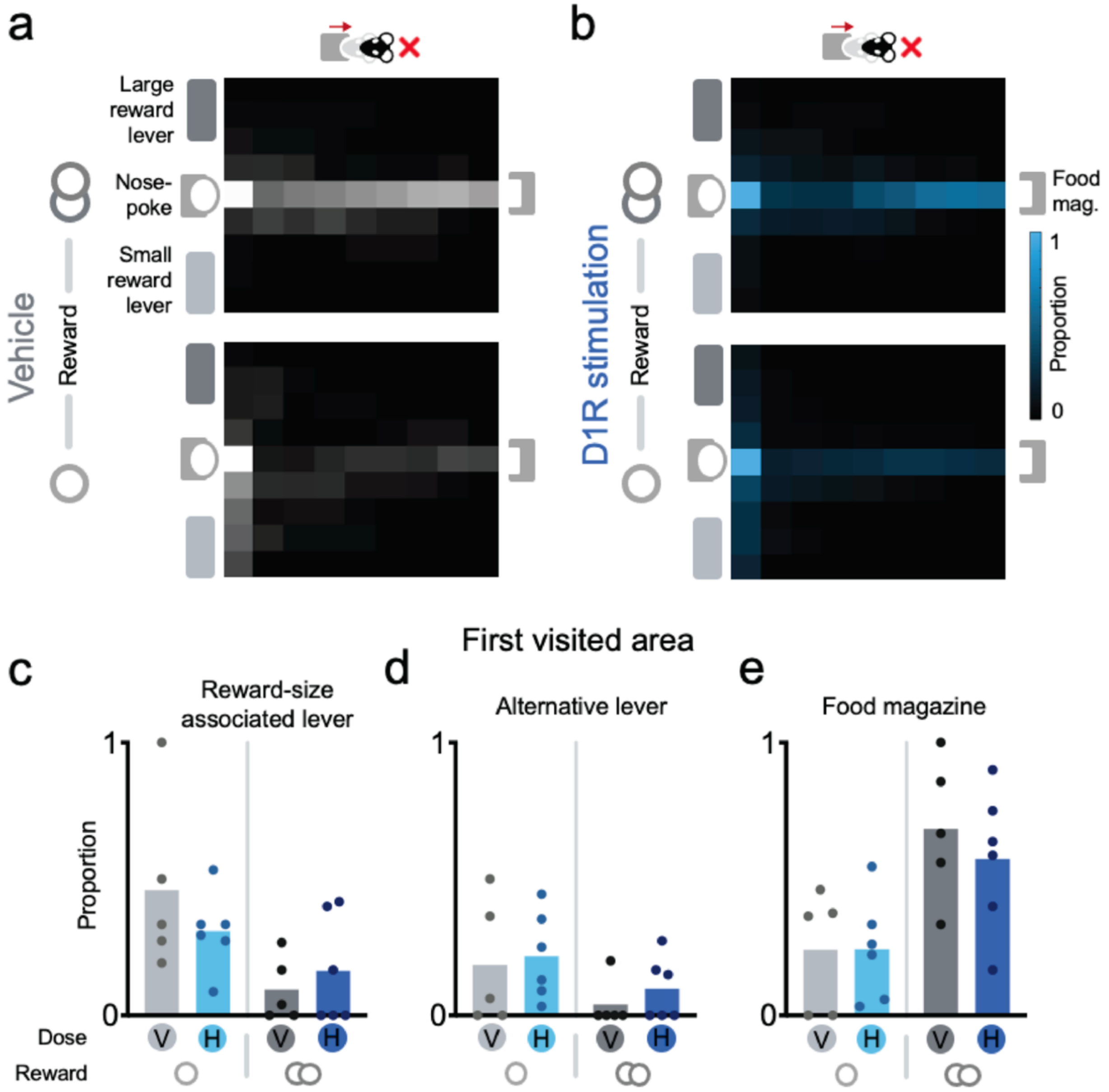

Supp. Fig. 3. The effects of intra-NAcC D1R stimulation (SKF-81297) in error No-Go trials. (a, b) Mean probability density across rats in small (lower) or large (upper) reward error No-Go trials when **(a)** on vehicle or **(b)** with intra-NAcC infusion of the D1R agonist. **(c-e)** Proportion of trials in which the first area of the operant chamber visited by the rats was **(c)** the reward size-associated lever, corresponding to the large reward lever on large reward No-Go trials and the small reward lever on small reward No-Go trials, **(d)** the alternative lever, and **(e)** the food magazine. Location pairwise comparisons: reward-size associated lever vs. food magazine: $p = .032$, alternative lever vs. food magazine: $p = .002$, reward-size associated lever vs. alternative lever: $p = .196$.

Supplementary figure 4

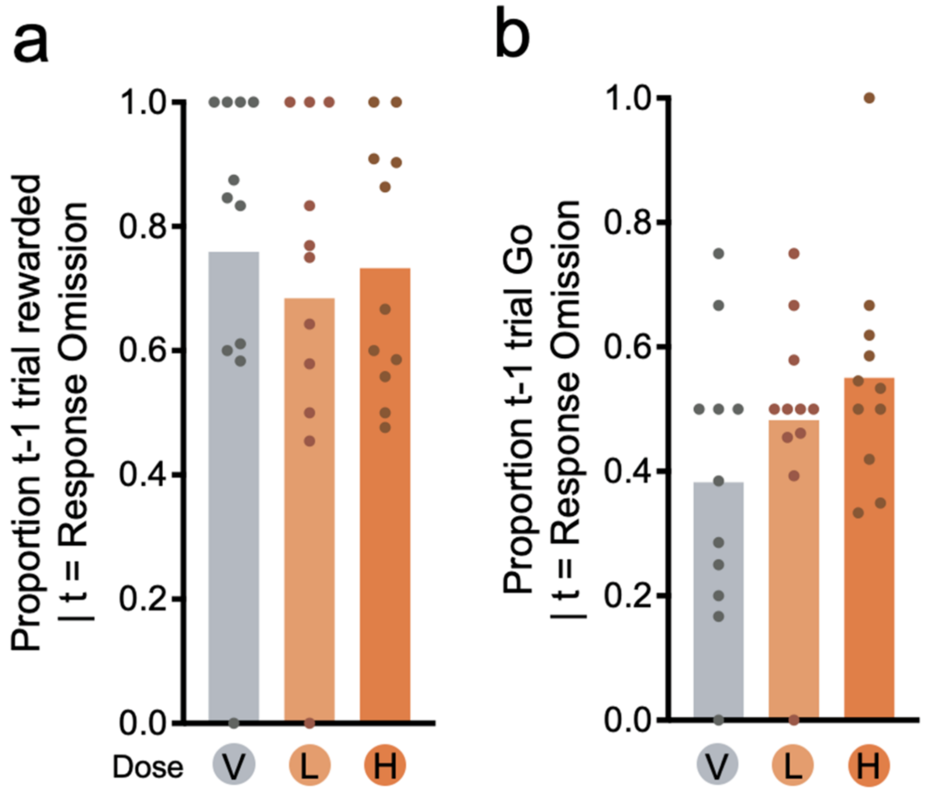

Supp. Fig. 4. The effects of intra-NAcC D1R blockade on trial-by-trial predictors of fluctuations in Go trial performance. V = vehicle, L = low dose, H = high dose. **(a)** The average proportion of Response Omission error trials preceded by a rewarded trial. **(b)** The average proportion of Response Omission error trials preceded by a Go trial.

Supplementary figure 5

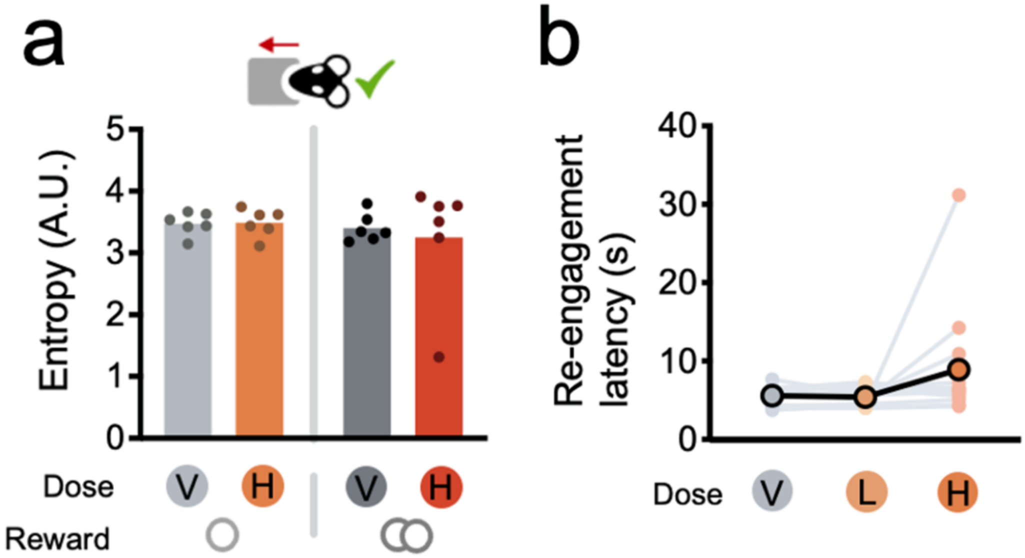

Supp. Fig. 5. The effects of intra-NAcC D1R blockade on additional metrics of engagement and noisiness. (a) Average entropy of animals moving from the nosepoke to the food magazine in successful No-Go trials. V = vehicle, H = high dose. Single circle indicates small reward condition, double circle indicates large reward condition. **(b)** Average time to attempt to initiate the next trial. V = vehicle, L = low dose, H = high dose.

Supplementary figure 6

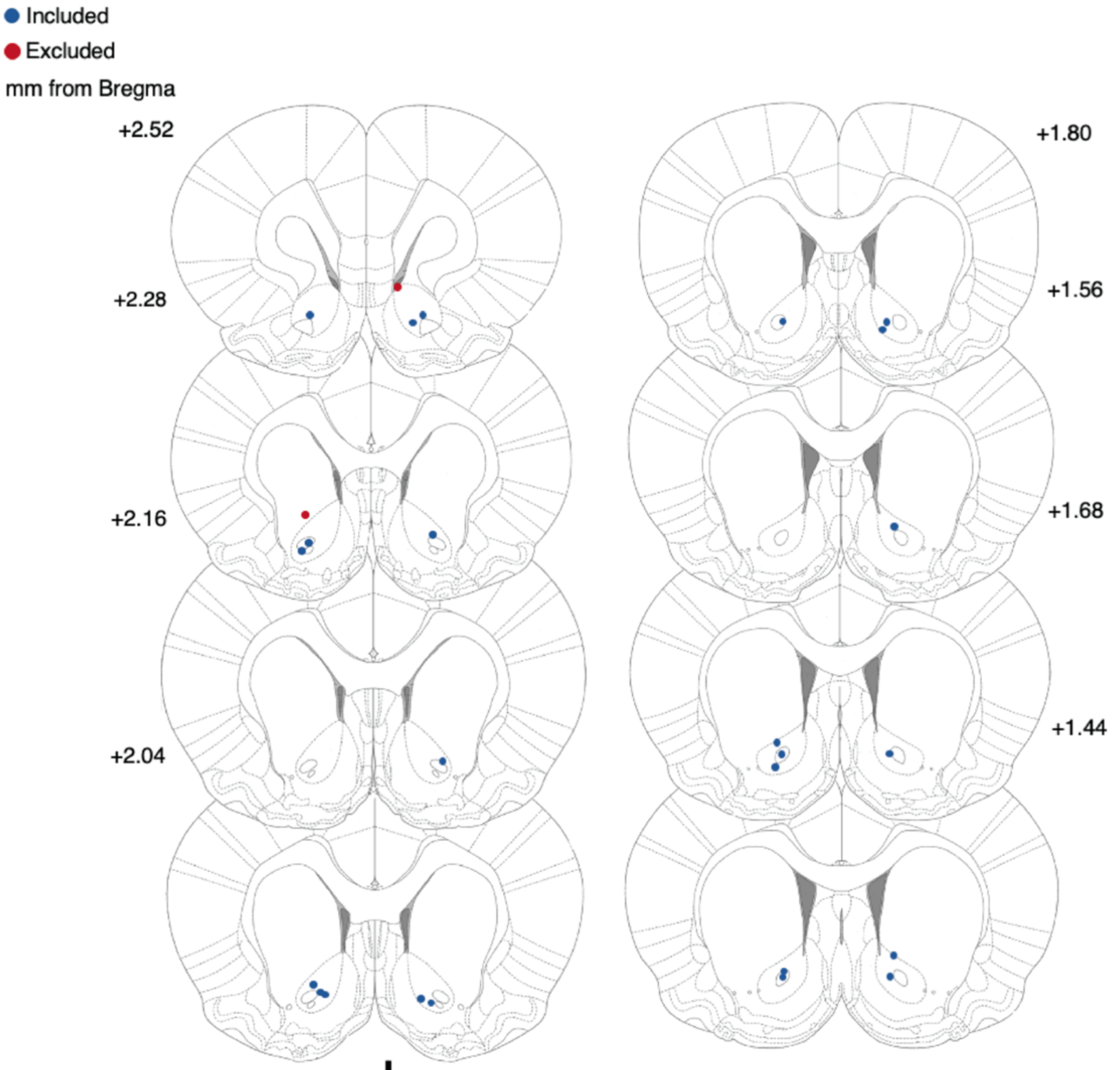

b

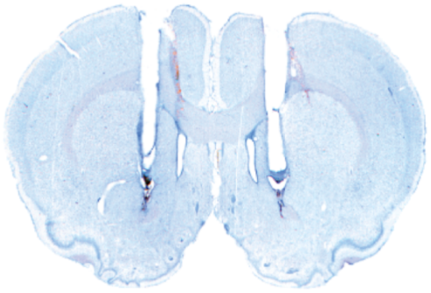

Supp. Fig. 6. Injector placement. (a) Schematic of cannula insertion locations in the NAcC (n = 14, all rats bilaterally implanted). Cannulae locations of included rats are marked in blue, excluded rats (n = 1) are marked in red. Numbers to the left of coronal sections indicate distance anterior to bregma (mm). Adapted from the atlas of Paxinos and Watson (2009). **(b)** Example photo scan of a perfused section showing bilateral injector lesion and guide cannulae placement.
